# Characterization of the mitogenomes for two sympatric breeding species in Recurvirostridae (Charadriiformes) and their phylogenetic implications

**DOI:** 10.1080/23802359.2017.1307704

**Published:** 2017-03-29

**Authors:** Chao Yang, Qing-Xiong Wang, Xue-Juan Li, Hong Xiao, Yuan Huang

**Affiliations:** a Shaanxi Institute of Zoology, Xi’an, China;; b School of Life Sciences, Shaanxi Normal University, Xi’an, China

**Keywords:** *Himantopus himantopus*, *Recurvirostra avosetta*, mitogenome, structure, phylogeny

## Abstract

Recurvirostridae is a family of Charadriiformes that displays an amazing amount of characterization at evolutionary level. The mitogenomes of *Himantopus himantopus* and *Recurvirostra avosetta* are 17,378 bp and 16,856 bp in size, respectively. Both two mitogenomes reveal the same gene order and genomic organization to that of typical avian mtDNA. The first conserved block with two interrupted poly-C and four long terminal repeats with 140 bp are present in *H. himantopus* control region. Phylogenetic analysis indicated that Recurvirostridae (*H. himantopus* and *R. avosetta*) has the closest relationship with Haematopodidae. We supported that Stercorariidea is a sister group to (Alcidae (Laridae, Sternidae)), and suggested that the status of *Larus vegae* should be further investigated.

Black-winged stilt (*Himantopus himantopus*) and Pied Avocet (*Recurvirostra avosetta*) belonging to Recurvirostridae, are widely distributed in shallow and brackish wetlands, feeding mainly on macroinvertebrates and both of their Northern populations making long-distance southwards migratory movements. The two species both have extremely large range and population size, and are evaluated as Least Concern (IUCN 2016), but their important habitat sites are losing and fragmentating in infrastructure development, pollution, human disturbance, and reduced river flows (del Hoyo et al. 1996; Kelin & Qiang 2006) and they may be threatened by future outbreaks of avian botulism and influenza (Hubalek et al. 2005; Melville & Shortridge 2006). The complete mitogenome is an important marker for studies related to taxonomy, biodiversity, and conservation (Anmarkrud & Lifjeld 2017). Using complete mitogenomes, one can also analyze nucleotide variation, obtain information on haplotypes, and elucidate current population structures of species (Yamamoto et al. 2005). Nevertheless, in terms of phylogenetics, the limited molecular data dampen the evolution and diversity studies in interspecific of Recurvirostridae.

Naturally dead *H. himantopus* and *R. avosetta* chicks were collected during the breeding season at Hongjian Nur (39°04′N, 109°53′E), Shaanxi, China. The specimens (proof number: H01, T01) were preserved in 100% ethanol and stored at −20 °C, and deposited in the animal specimens museum of Shaanxi Institute of Zoology, Xi’an, China. The mitogenomes were determined after PCR amplification, sequencing, and annotation based on previously published sequence (Yang et al. 2012).

The complete mitogenomes of *H. himantopus* (Genbank: KY623656) and *R. avosetta* (Genbank: KY623657) are 17,378 bp and 16,856 bp in length, and with the base composition A + T are 55.6% and 55.3%, respectively. The 13 PCGs of *H. himantopus* and *R. avosetta* are similar to that observed for other charadriiformes with *ATP8* and *ND5* being the shortest and longest genes, respectively. The typical ATN (ATG, ATT, or ATC) start codons are present in 11 of the 13 *H. himantopus* and *R. avosetta* PCGs, with the exception of the *COI* and *ND5* in two species, which utilize GTG as start codons. Four types of stop codons are used, including TAA, AGG, and TAG for most of the genes, and an incomplete stop codon T–– for *COX3*, *ND2,* and *ND4* in two species. The *ND3* genes of *H. himantopus* and *R. avosetta* are all with an extra C nucleotide (Yang et al. 2016).

The *srRNA* and *lrRNA* in *H. himantopus* and *R. avosetta* are 977, 1591 bp and 967, 1594 bp, respectively. All the tRNA genes in two species that can fold into typical cloverleaf secondary structures except for *tRNA^Ser^(AGY)*, which lack the DHU arm. The control regions are 1818 bp and 1293 bp in length in *H. himantopus* and *R. avosetta*. Especially, in *H. himantopus*, the first conserved block (5′-CCCCCCCCCTACCCCCCCATGCATATCGCTACCCCCCCTACCCCCCC-3′; bp positions 15,583–15,629) in domain I with two interrupted poly-C sequences, and four consecutive repeats with 140 bp (bp positions 16,566–17,125) and 35 simple sequence repeats 5′-CAAA-3′ (bp positions 17,236–17,375) are present in its 3′ end of the control region. Only 26 repeats with 5′-CAAA-3′ (bp positions 16,746–16,849) are existed in *R. avosetta*.

To validate the phylogenetic positions of *H. himantopus* and *R. avosetta* (Recurvirostrida), Bayesian Inference (BI) and Maximum likelihood (ML) methods were employed to analyze the 28 mitogenomes PCGs to construct the phylogenetic trees. BI tree was constructed using MrBayes ver. 3.2.2 (Ronquist et al. 2012) under the best partitioned scheme and optimal model analyzed in Partitionfinder v1.1.1 (Lanfear et al. 2012) (Models GTR + I + G and GTR + G). ML analysis was performed using RAxML, and the robustness of the phylogenetic result was tested through bootstrap analysis with 1000 replicates (Li et al. 2016). *Grus vipio* and *Gallirallus philippensis* were selected as an outgroup. The topological structure of BI tree was the same with that of ML tree (data no show), except Stercorariidea in BI tree was a sister group to (Alcidae (Laridae, Sternidae)) (bootstrap value 1.00), but in ML tree it is closely related to Alcidae, then sister to Laridae and Sternidae ((Stercorariidea, Alcidae) (Laridae, Sternidae)) (bootstrap value 0.82). The phylograms obtained from BI and ML were all strongly supported that Recurvirostridae has the closest relationship with Haematopodidae (Thomas et al. 2004b; Fain & Houde 2007; Livezey 2010). Extraordinarily, the *Larus vegae* was more primitive and located in the root of the tree, but not belonged to the branch of Laridae (Figure 1).

**Figure 1.**
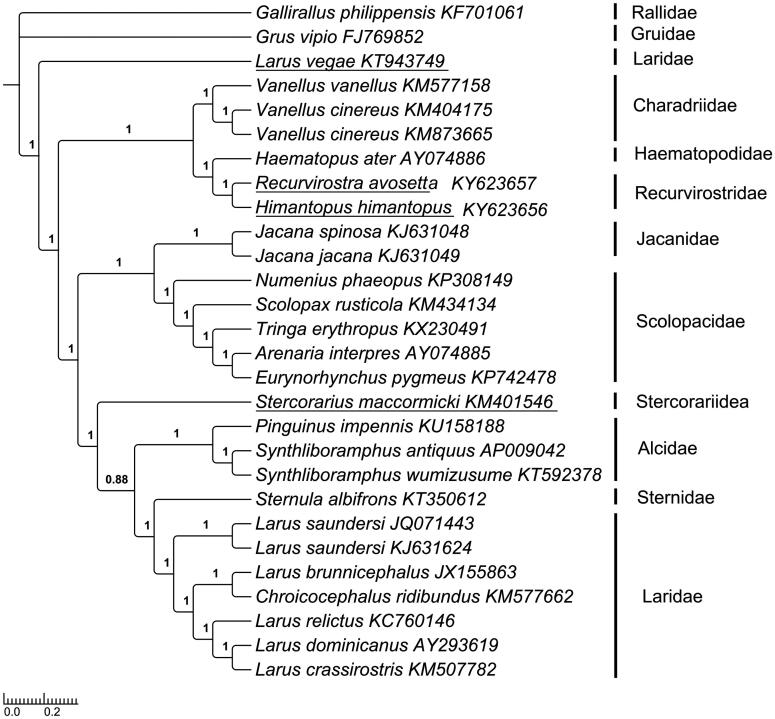
Topology of Bayesian tree for 28 species based on mitogenome PCGs sequences. GenBank accession numbers are indicated following species name. (Numbers on nodes are bootstrap values).
